# Development of rhesus macaque enteroids and enteroid-derived cell lines for enteric calicivirus infections

**DOI:** 10.1128/jvi.00745-26

**Published:** 2026-09-28

**Authors:** Tibor Farkas, Zeinab R. Aboezz, Mafruha Marjia, Jianrong Li

**Affiliations:** 1 Department of Veterinary Pathobiology, College of Veterinary Medicine and Biomedical Sciences, Texas A&M University 14736, College Station, Texas, USA; 2 Department of Virology, Faculty of Veterinary Medicine, Benha University, Toukh, Egypt; 3 Department of Veterinary Integrative Biosciences, College of Veterinary Medicine & Biomedical Sciences, Texas A&M University 14736, College Station, Texas, USA; University of Michigan Medical School, Ann Arbor, Michigan, USA

**Keywords:** rhesus macaque, intestinal stem cell-derived enteroids, rhesus enteric calicivirus

## Abstract

Recoviruses (ReCV) serve as valuable surrogates for fastidious human noroviruses (HuNoV). Although HuNoVs can be propagated in small intestinal enteroids, this system has several limitations, including low replication efficiency and an inability to sustain continuous propagation for generating infectious virus stocks beyond certain strains, such as GII.3. In contrast, ReCVs can be efficiently propagated in conventional cell lines (e.g., LLC-MK2), which has significantly advanced understanding of their biological characteristics. As a result, ReCVs are widely used as HuNoV surrogates. However, a physiologically relevant ReCV propagation system that closely mimics HuNoV enteroid cultures has not yet been described. Here, we report the establishment of intestinal stem cell-derived rhesus macaque enteroid lines representing both the small intestine and colon, along with two enteroid-derived cell lines. We further characterize these systems and evaluate their susceptibility to infection with three diverse ReCV isolates. Both small intestine- and colon-derived enteroids, as well as their corresponding cell lines, supported robust ReCV infection. The enteroids contained all major intestinal cell types. The duodenum-derived cell line comprised enteroendocrine cells (chromogranin A) and absorptive enterocytes (villin), whereas the colon-derived cell line included enteroendocrine cells and goblet cells (MUC2). Importantly, both cell lines formed tight barriers (transepithelial electrical resistance [TEER] > 3,000 Ω·cm²), making them far superior to LLC-MK2 cells (TEER ~ 100 Ω·cm²) for studies of viral entry and pathogenesis. The rhesus enteroids and intestinal cell lines developed in this study will enable comparative studies of HuNoV and ReCV replication in a physiologically relevant system and may have direct applications for culturing HuNoVs.

**IMPORTANCE:**

Human intestinal stem cell-derived enteroids have enabled the propagation of human noroviruses; however, this system has several important limitations, including high cost, low replication efficiency, and (except for a recent report for GII.3 human norovirus) the inability to sustain continuous virus propagation for the generation of infectious virus stocks. As a result, research still relies on human norovirus-containing clinical samples as the primary virus source. Here, we developed host-specific rhesus macaque enteroid cultures and two enteroid-derived cell lines that support robust recovirus replication. Studying recovirus replication in rhesus enteroids may help overcome key limitations of the human norovirus enteroid cultures. Moreover, because both natural and experimental human norovirus infections have been described in rhesus macaques, the rhesus enteroids and enteroid-derived cell lines described in this study may be directly applicable to human norovirus cultivation.

## INTRODUCTION

Human noroviruses (HuNoV) are the leading cause of acute gastroenteritis worldwide (1–3). Although the prototype Norwalk virus was first reported in 1972, HuNoV research was long hampered by the inability to culture the virus *in vitro* (4). For decades, norovirus research relied heavily on surrogate caliciviruses that could be cultivated in cell culture. In 2016, a HuNoV cultivation system was reported using human small intestinal stem cell-derived enteroid cultures (5). LGR5^+^ intestinal stem cells and their essential growth factors—Wnt3A, R-spondin, Noggin, and EGF—were identified in 2007, laying the foundation for the development of *ex vivo* intestinal organoid cultures, often referred to as “mini-guts” (6). These organoids can be generated either from isolated crypts or purified LGR5^+^ stem cells, resulting in enteroids composed solely of epithelial cells, or from induced pluripotent stem cells, producing organoids that include both epithelial and mesenchymal components. Both systems recapitulate the cellular diversity of the native intestine, including stem cells, Paneth cells, goblet cells, enterocytes, M cells, and enteroendocrine cells. Compared to traditional immortalized cell lines, these organoid models provide a more physiologically relevant and genetically stable platform for research. Enteroids support the replication of several HuNoV strains from different genogroups and genotypes. Bile is an essential requirement for the replication of most HuNoV strains. Although GII.4 strains can replicate in the absence of bile, their replication is significantly enhanced upon bile supplementation (7). Despite providing a long-awaited *in vitro* replication model for HuNoV research, the enteroid system has several notable limitations (8). These include low replication efficiency, the requirement for a high initial multiplicity of infection (MOI), and the inability to sustain continuous virus propagation to generate high-titer infectious HuNoV stocks. Consequently, infection studies continue to rely on high-titer HuNoV-containing clinical samples, which are difficult to obtain and inherently limited. Moreover, not all HuNoV-positive stool samples—including those with high viral titer—support productive replication in enteroid cultures (9).

During the revision of this manuscript, enhanced replication and sustained passaging of a GII.3 human norovirus were reported in genetically modified, originally non-secretor enteroid lines expressing FUT2 via a lentiviral vector (J4FUT-KI and J8FUT-KI), in combination with treatment using TAK-779, a CXCR3/CCR5/CCR2 antagonist. In contrast, replication of pandemic GII.4 human noroviruses remained unaffected under the same conditions, highlighting substantial differences among enteroid lines and human norovirus genotypes in the mechanisms regulating and controlling viral replication in intestinal enteroids (10). Additionally, serial passaging of a GII.17 human norovirus in the unmodified jejunal HIE J2 enteroid line following treatment with ruxolitinib and TAK-779 has also been described (11).

While the enteroid system continues to overcome several constraints in human norovirus research, important discrepancies remain compared with natural infection, including the extremely low minimum infectious dose and the high levels of viral shedding observed in infected individuals.

Recoviruses (ReCVs) are recognized as one of the most relevant surrogate viruses for human HuNoV research. This is primarily due to their ability to propagate efficiently in conventional cell cultures (e.g., LLC-MK2 cells), which has significantly advanced our understanding of various aspects of ReCV biology, including host susceptibility determinants, serotypic diversity, and other key characteristics (12–14).

Although ReCVs represent a novel genus within the *Caliciviridae* family, they are evolutionarily closely related to noroviruses and have retained several biological features of HuNoVs, likely inherited from a common ancestor. These include a primate host, broad genetic and antigenic diversity, enteric tropism, clinical manifestation as acute gastroenteritis, and the use of histo-blood group antigens (HBGAs) and sialic acid as attachment factors, along with strain-specific dependency on these molecules (12).

Moreover, while the entry receptor(s) for HuNoVs remain unidentified, the entry receptor and strain-specific host determinants of susceptibility (involving attachment and entry receptors) for ReCVs have been recently characterized (13, 14).

Despite their utility as a HuNoV surrogate, a physiologically relevant propagation system for ReCVs that closely mirrors the HuNoV enteroid model has yet to be established. Both HuNoVs and ReCVs are enterotropic, and while enteroids comprise all major intestinal cell types, LLC-MK2 cells are transformed rhesus kidney epithelial cells. Here, we report the development of rhesus macaque enteroid lines representing both the small and large intestine, as well as two enteroid-derived cell lines, and their characterization and evaluation in ReCV infection studies.

## RESULTS

### Establishment of rhesus macaque enteroid lines

The crypt isolation and enteroid culture protocols described in Materials and Methods were effective for generating both small intestinal (duodenum, jejunum, and ileum) and large intestinal enteroids (colon and rectum) from multiple rhesus macaques of varying ages and both sexes (Table 1).

**TABLE 1:** Rhesus macaque enteroid cell lines used in this study

ID	Place of origin	Sex	Age	Intestinal section^*a*^	Reason for euthanasia
**NC67**	TNPRC	Male	9 mo (infant)	**D** and **C**	Colitis
**NE49**	TNPRC	Male	8 mo (infant)	D and **C**	Colitis
**M1**	SNPRC	Male	7 mo (infant)	S, D, J, **I**, and C	Chronic wasting

^*a*^ S, stomach; D, duodenum; J, jejunum; I, ileum; C, colon. IDs and sections used in the study are bold.

**Fig 1:**
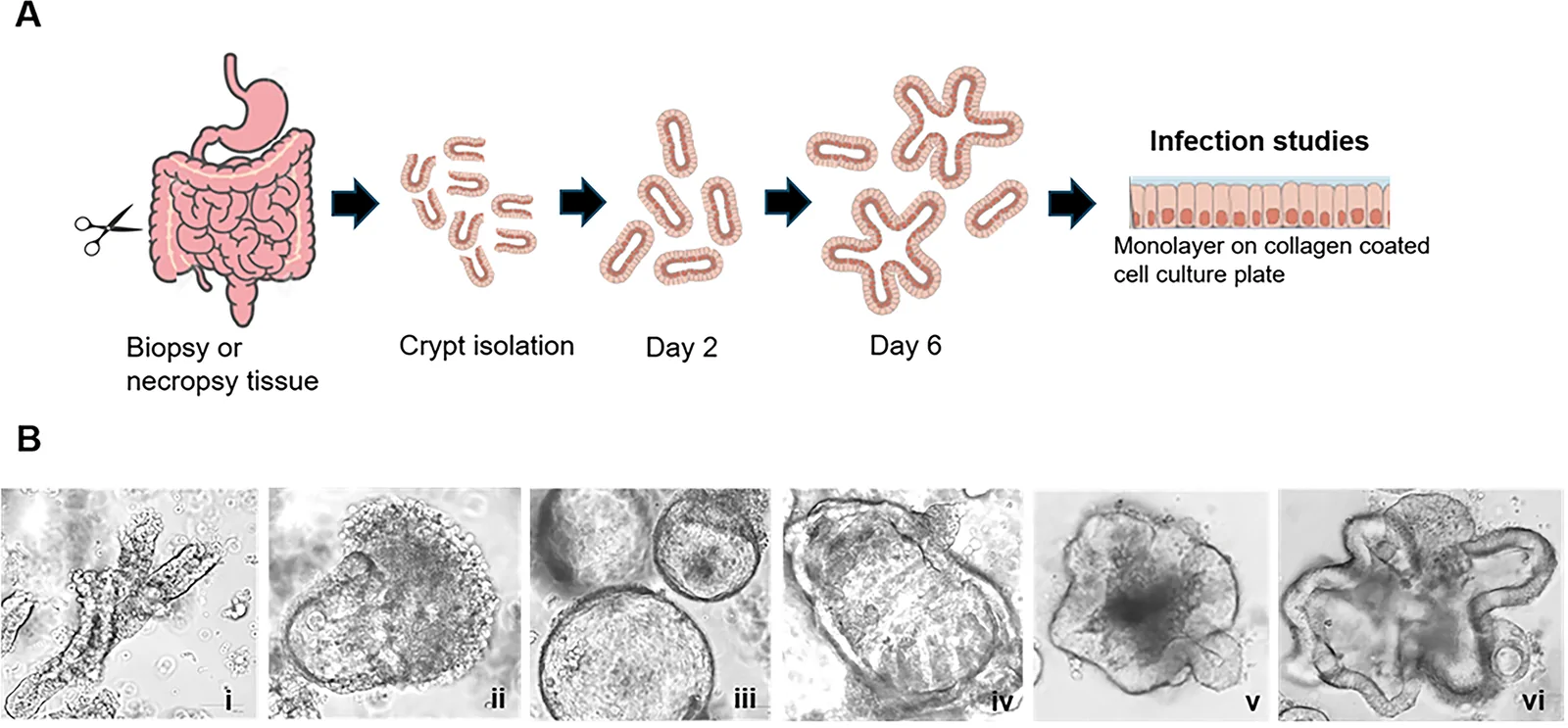
(**A**) Schematic overview of the development of intestinal stem-cell–derived enteroids. (**B**) Representative images illustrating developmental stages of rhesus macaque enteroids grown in Matrigel domes: (i) isolated crypts, (ii) crypts expelling cellular debris, (iii) early enterospheres, (iv) maturing enterospheres, and (v and vi) enteroids displaying emerging budding structures.

Growth rates varied among individual animals and intestinal segments, though the underlying causes of these differences remain unclear. Typically, Matrigel-embedded crypts began to swell and expel cellular debris through the apical opening within 12 h of plating. By 24 h, the openings had closed, and the structures transitioned into spherical enterospheres (Fig. 1B, i–iii). By day 4, these enterospheres became denser and less spherical, with thicker walls composed of columnar epithelial cells. Between days 6 and 8, they matured into enteroids characterized by budding protrusions and folded structures surrounding a central lumen, indicative of expanding crypt-like domains (Fig. 1B, iv–vi).

We successfully established over 15 enteroid lines from four rhesus macaques, encompassing the entire intestinal tract from the stomach to rectum. Each line demonstrated robust growth and was expanded for at least 25 passages. Only small intestine- and colon-derived enteroid lines were used in this study (Table 1).

### Cell type composition of rhesus macaque enteroids

To confirm the presence of the major intestinal cell types in rhesus macaque enteroids, we used commercially available antibodies targeting established intestinal cell markers, including E-cadherin and EpCAM (epithelial cells), villin and sucrase-isomaltase (enterocytes), chromogranin A (enteroendocrine cells), MUC2 (goblet cells), lysozyme-C (Paneth cells), and LGR5 (intestinal stem cells). Enteroids were analyzed using both immunofluorescence microscopy and western blotting.

**Fig 2:**
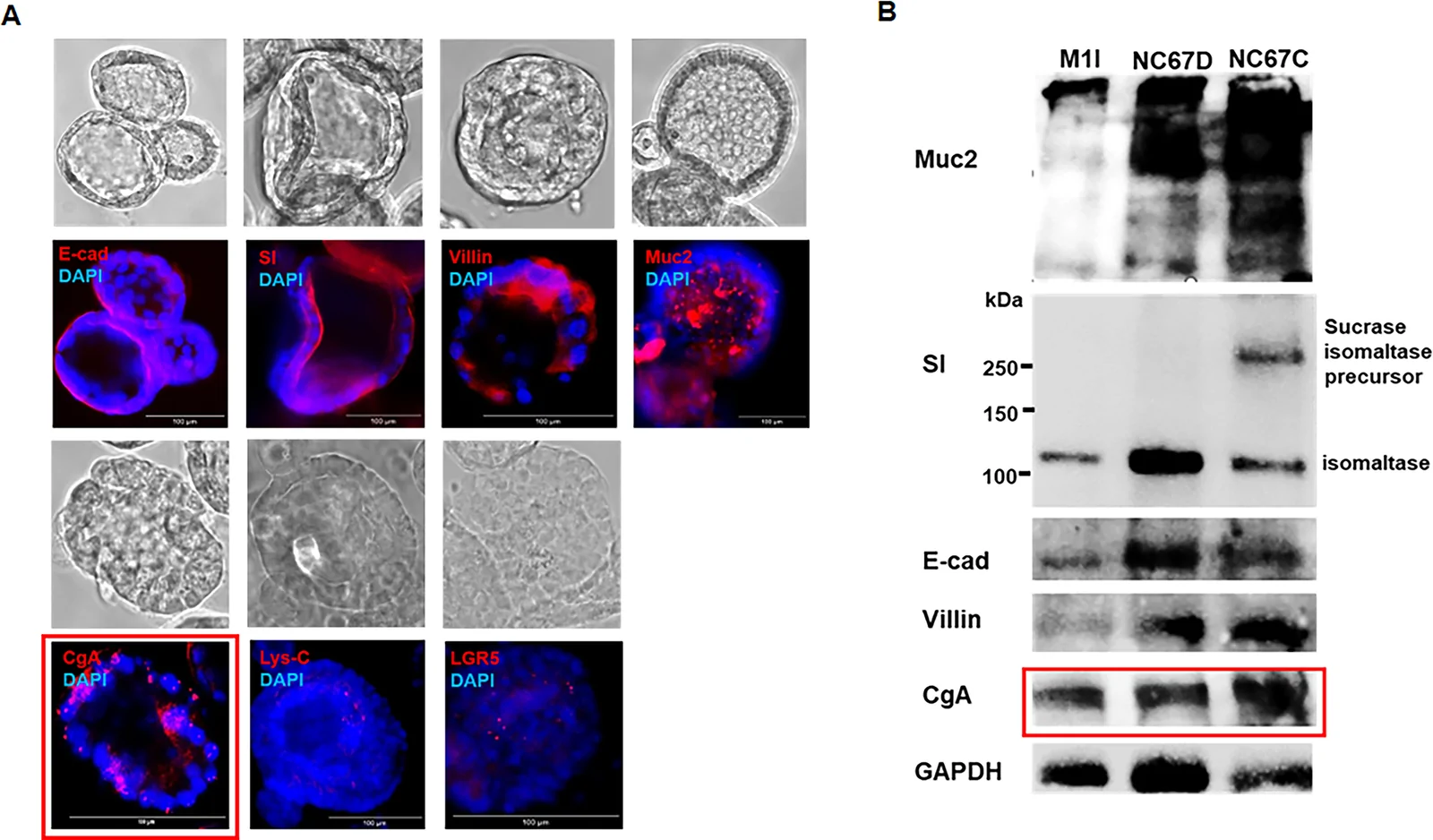
Cell type composition of rhesus enteroids. (**A**) Immunofluorescence imaging of NC67D (duodenum). Scale bar = 100 µm. (**B**) Western blot analysis of cell type markers in M1I (ileum), NC67D (duodenum), and NC67C (colon). Markers: E-cadherin: epithelial cells, sucrase-isomaltase and villin: brush border of absorptive enterocytes, Muc2 = goblet cells, chromogranin A (CgA) = enteroendocrine cells, lysozyme-C (Lys-C) = Paneth cells, and LGR5 = stem cells. Based on inconsistent staining patterns with the CgA antibody (sc-393941, Santa Cruz Biotech) and confocal imaging (see Fig. 4), the CgA staining (red box) was repeated using a second CgA antibody (MA5-13096, Invitrogen) (see Fig. 5).

Immunofluorescence staining confirmed the presence of all major intestinal cell types in the rhesus enteroids, including enterocytes, enteroendocrine cells, goblet cells, intestinal stem cells, and Paneth cells (Fig. 2A). Western blot analysis confirmed the expression of most cell type-specific protein markers but did not detect LGR5 or lysozyme-C (data not shown; Fig. 2B). This may reflect the lower number of intestinal stem cells and Paneth cells in the enteroids or limitations of the monoclonal antibodies used for detection under reducing conditions. Nevertheless, the ability to expand and serially culture all enteroid lines supports the presence of intestinal stem cells and Paneth cells.

### ReCVs replicate in both small intestine- and colon-derived enteroid monolayers

Three genogroup I (GI) ReCV isolates, representing three distinct genotypes/serotypes and HBGA binding types (12), were evaluated for their ability to replicate in rhesus macaque enteroid monolayers. All three strains require the coxsackievirus and adenovirus receptor (CAR) for cell entry. In addition, Tulane virus can utilize type A and B HBGAs or sialic acid for attachment, whereas ReCV-FT285 attachment is sialic acid independent and requires type A or B HBGAs. In contrast, ReCV-FT7 attachment is HBGA-independent and relies on sialic acid (13, 14).

**Fig 3:**
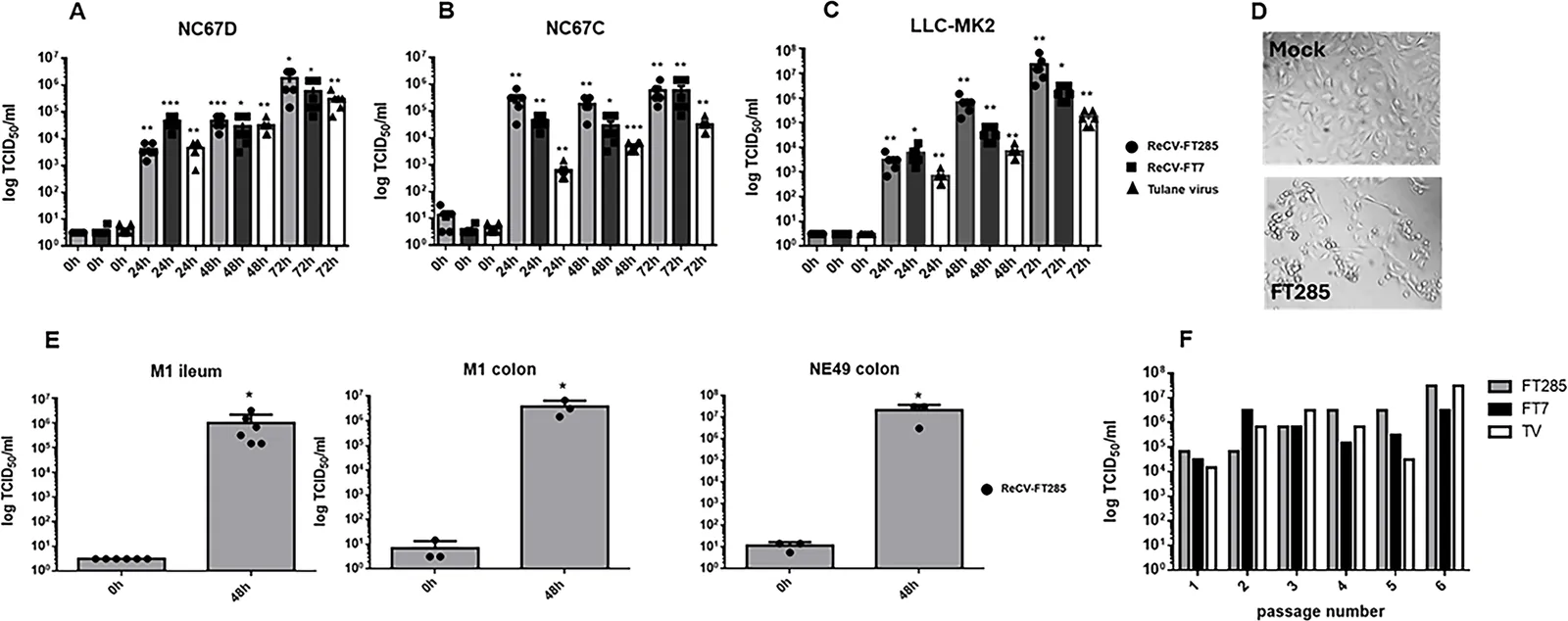
ReCV infection in rhesus macaque enteroid monolayer cultures. Monolayers were infected with each virus at an MOI of 1 for 1 h, washed three times, and overlaid with fresh culture media. Samples were collected at the indicated time points post-infection (pi), and infectious virus titers (TCID_50_) were determined by 10-fold serial dilutions in 96-well plates seeded with LLC-MK2 cells. Both duodenum (**A**) and colon-derived (**B**) enteroid monolayers supported robust ReCV replication, yielding end-point titers comparable to those observed in LLC-MK2 cells (**C**). (**D**) Representative images of mock- and ReCV-FT285-infected NC67D monolayers at 48 hpi. Infected cultures exhibit pronounced cytopathic effects, including cell rounding and detachment, which were noticeable by 24 hpi and intensified over time. (**E**) Enteroid monolayers derived from both the small intestine and colon of multiple rhesus macaque donors supported productive ReCV infection (data shown for ReCV-FT285). (**F**) ReCV isolates were serially passaged in NC67D enteroid monolayers by transferring 10 µL of a 100 µL culture to fresh wells every 72 h post-infection. All three ReCV strains remained infectious through at least six serial passages without reduction in viral titers. Data represent the mean ± SD of at least three biological replicates. *P* ≤ 0.05; *P* ≤ 0.01; **P ≤ 0.001.

Both rhesus macaque duodenum- (NC67D) and colon-derived (NC67C) enteroid monolayers supported robust replication of all three ReCV isolates (Fig. 3A and B), and peak virus titers reached comparable levels to those obtained in LLC-MK2 cells, the only commercially available cell line in which ReCVs can be successfully isolated from clinical samples (15, 16) (Fig. 3C). Infection resulted in pronounced cytopathic effects (CPEs) that intensified by post-infection (pi) time and were characterized by cell rounding, detachment, and lysis (Fig. 3D). Infection and replication efficiency were similar across several donor animals and intestinal segments (Fig. 3E). Although replication of several HuNoV strains in enteroid cultures has been reported, continuous passaging has remained challenging. Recently, sustained passaging of GII.3 and GII.17 HuNoVs was reported in the presence of chemokine receptor inhibitor TAK-779 or TAK-779 and ruxolitinib (JAK1/2 inhibitor), respectively (10, 11). Therefore, we evaluated whether ReCVs can be continuously subcultured in enteroid monolayer cultures. All three ReCV isolates could be serially passed at least six times without the need to inhibit innate immune responses (Fig. 3F).

### Enteroid-derived cell lines

**Fig 4:**
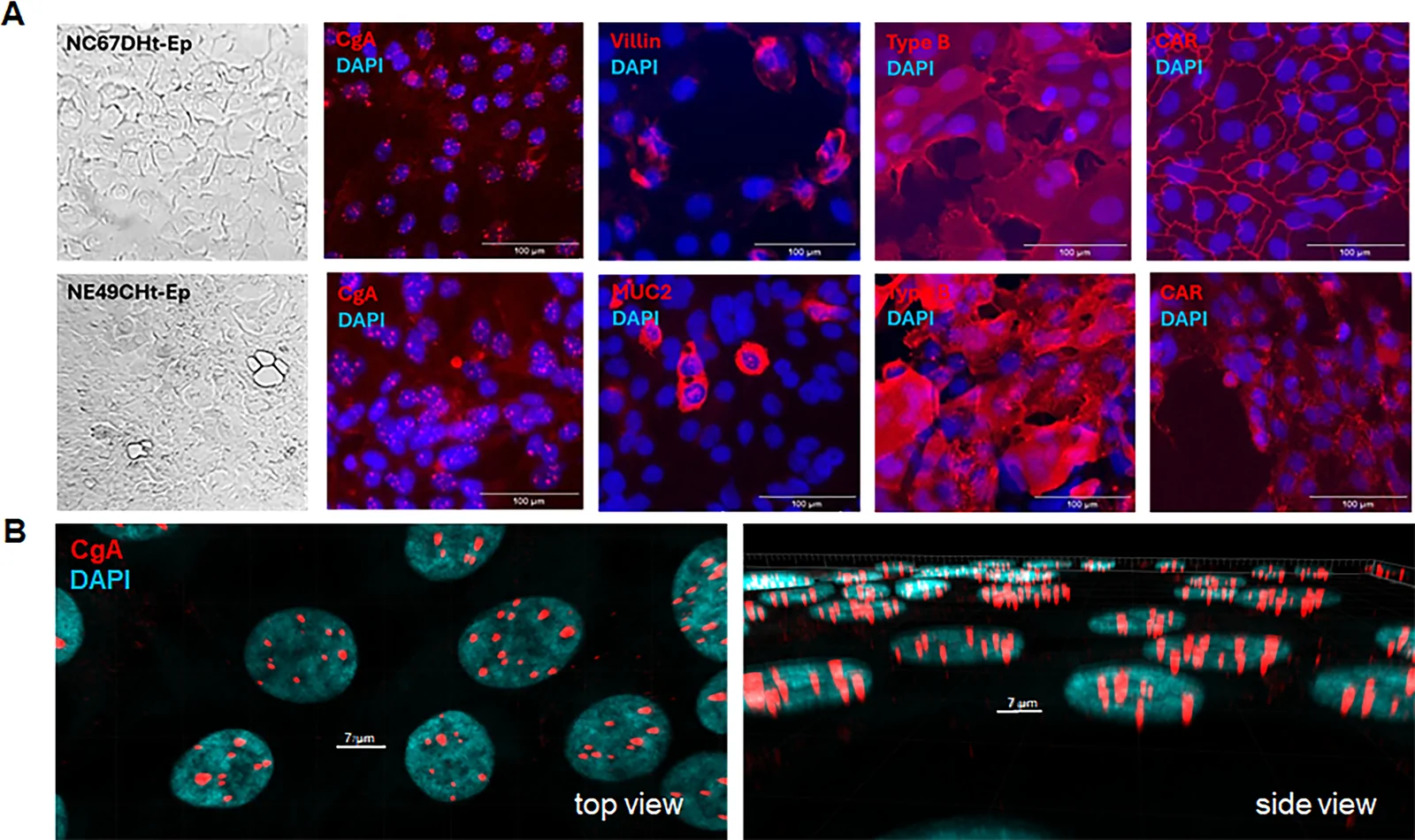
Cell type composition of rhesus enteroid-derived cell lines. (**A**) Both cell lines exhibit typical epithelial morphology. Immunofluorescence staining for cell type-specific markers revealed the presence of enteroendocrine cells (chromogranin A) and absorptive enterocytes (villin) in the NC67DHt-Ep cell line, while the NE49CHt-Ep cell line contained enteroendocrine cells (chromogranin A) and goblet cells (MUC2). Additionally, both cell lines expressed CAR and type B HBGA, the two major susceptibility determinants for ReCV infection. Scale bar 100 μm. (**B**) Confocal images of the NC67DHt-Ep cell line stained with anti-chromogranin A mouse monoclonal antibody (sc-393941, Santa Cruz Biotech) were acquired using an Olympus FV4000 laser-scanning confocal microscope with a 60× oil-immersion objective. Maximum-intensity projections of *z*-stack images were reconstructed in three dimensions (3D). Scale bar 7 μm. The images show nuclear localization of the signal, whereas CgA is expected to be localized in the cytoplasm, indicating that this antibody is not specific for CgA.

We evaluated the feasibility of generating enteroid-derived epithelial cell lines using lentiviral vectors encoding hTERT. Transduced monolayers were passaged every 5–7 days with the goal of expanding cultures from 96-well plates to flasks. Most attempts were unsuccessful, as cultures either ceased proliferating or lost epithelial characteristics, likely due to epithelial–mesenchymal transition (EMT). However, two lines were successfully expanded for over 30 passages without morphological changes, retaining a cobblestone-like morphology characteristic of epithelial cells. These lines were designated NC67DHt-Ep (duodenum) and NE49CHt-Ep (colon), respectively (Fig. 4A).

Immunofluorescence staining confirmed the expression of CAR and type B HBGA in both cell lines that confer susceptibility to ReCV infection. In the NC67DHt-Ep cell line, the majority of cells expressed CgA, and a substantial fraction were positive for villin (Fig. 4A). In contrast, we did not detect cells positive for LGR5, lysozyme-C, MUC2, or sucrase-isomaltase (data not shown). In the NE49CHt-Ep cell line, most cells stained positive for CgA, but a significant number of cells displayed very strong MUC2 staining (Fig. 4A).

Closer examination of the CgA staining raised concerns about the specificity of the anti-chromogranin A monoclonal antibody used (sc-393941, Santa Cruz Biotech). First, in Fig. 2, the number of CgA-positive cells in the enteroids was markedly higher than the 1%–4% of enteroendocrine cells typically observed under physiological conditions. Second, in Fig. 4, the signal appeared to localize to the nucleus rather than the cytoplasm. Confocal microscopy further confirmed this nuclear staining, indicating that this antibody recognized a nuclear protein, rather than CgA (Fig. 4B).

### Re-evaluation of chromogranin A expression

**Fig 5:**
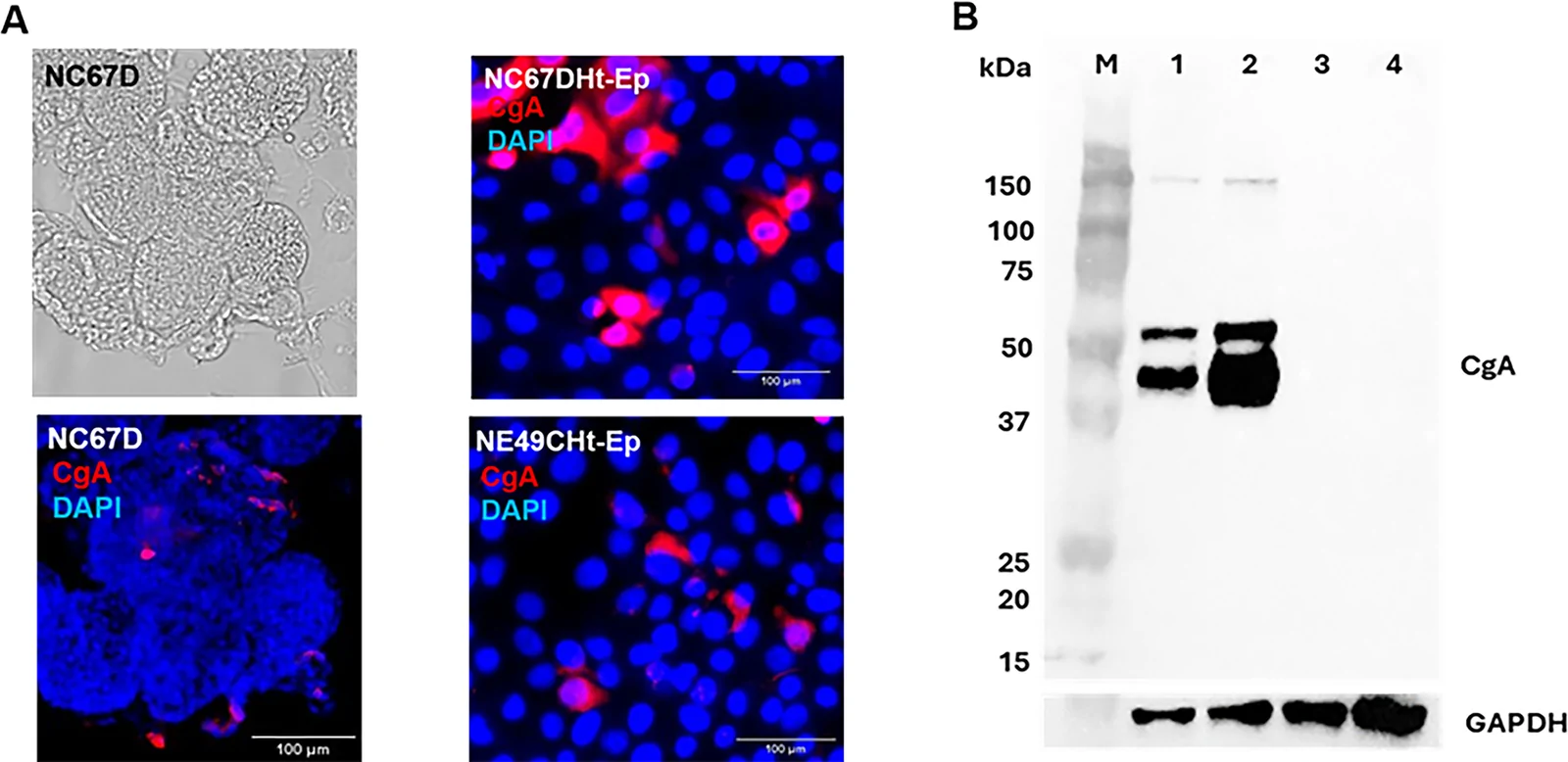
Re-evaluation of chromogranin A expression using mouse anti-chromogranin A monoclonal antibody (MA5-13096, Invitrogen). (**A**) Immunofluorescence staining of chromogranin A in NC67D (duodenum) enteroids and in enteroid-derived cell lines. Scale bar = 100 µm. (**B**) Western blot analysis of chromogranin A expression. Lane 1, NC67D enteroid; lane 2, NC67DHt-Ep cell line; lane 3, Vero cells; lane 4, LLC-MK2 cells.

To determine the presence of enteroendocrine cells in the enteroids and cell lines, we repeated immunofluorescent staining and western blot analysis using a different anti-chromogranin A monoclonal antibody (MA5-13096, Invitrogen). This antibody yielded a reduced number of IF-positive cells in the enteroids (Fig. 5A) and showed strong cytoplasmic staining in the cell lines (Fig. 5B). Antibody specificity was verified by the detection of a ~48 kDa protein band in the enteroid and cell line lysates, which was absent in Vero and LLC-MK2 lysates. Additionally, a secondary band (~52 kDa) was observed in the intestinal cell lysates, likely corresponding to a posttranslationally modified form of CgA (e.g., glycosylation).

Together, these results indicate that the NC67DHt-Ep cell line is likely composed of enteroendocrine cells and absorptive enterocytes, whereas the NE49CHt-Ep cell line contains enteroendocrine cells and goblet cells.

### ReCV replication in enteroid-derived epithelial cell lines

**Fig 6:**
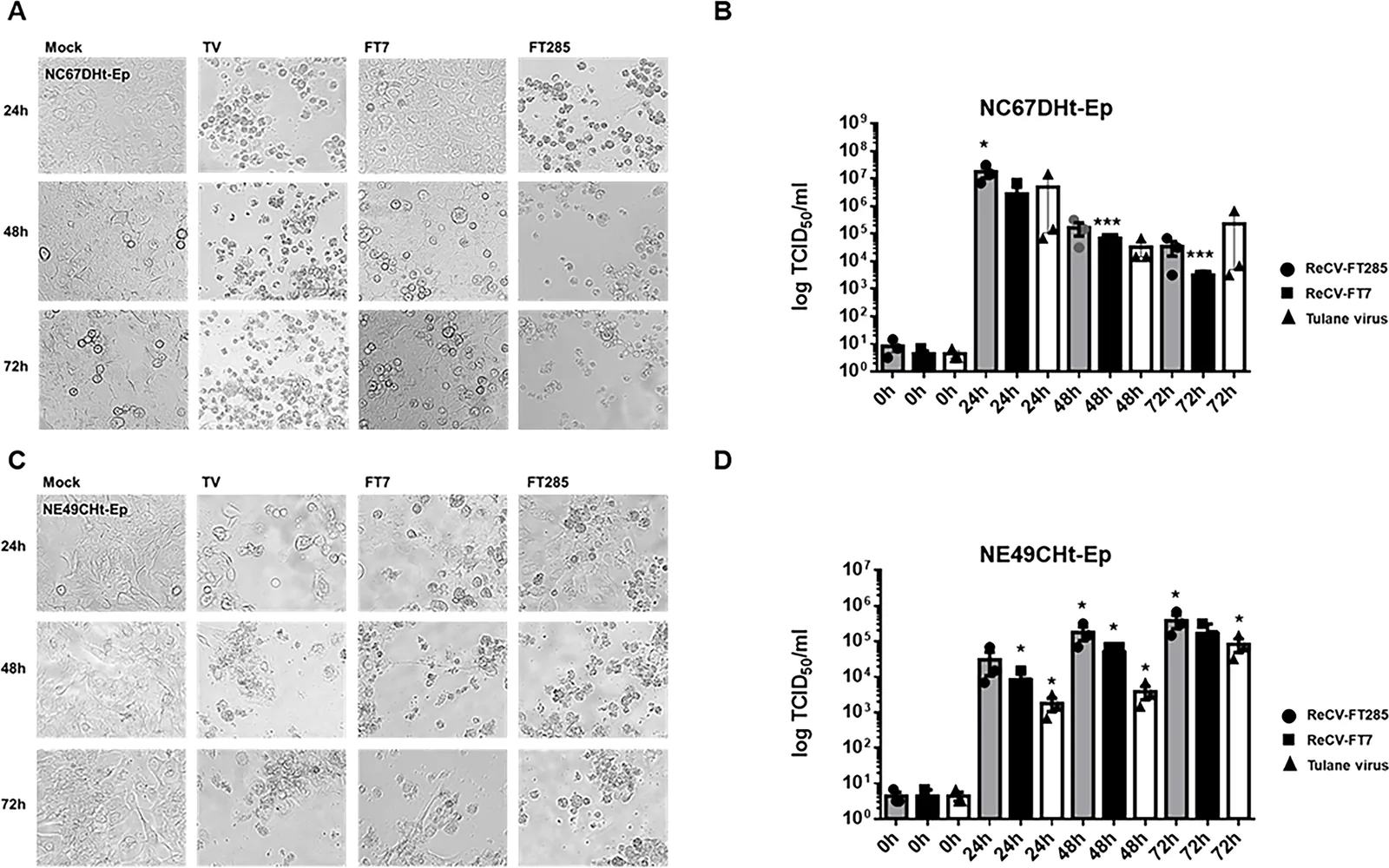
ReCV replication in enteroid-derived cell lines. Monolayers were infected with ReCVs at an MOI of 1. Morphological changes were monitored every 24 h post-infection (**A and C**), and viral yields were determined by titration in LLC-MK2 cells (**B and D**). Data represent the mean ± SD of three biological replicates. *P* ≤ 0.05; *P* ≤ 0.01; **P ≤ 0.001.

Both cell lines supported robust ReCV infection, accompanied by pronounced CPE. In NC67DHt-Ep cultures infected with TV or ReCV-FT285, CPE progressed rapidly, resulting in near-complete destruction of the monolayer by 24 h pi. ReCV-FT7-infected NC67DHt-Ep monolayers exhibited markedly slower CPE progression, with an almost confluent monolayer remaining visible beneath the detached cells even at 72 hpi (Fig. 6A). After the initial peak at 24 hpi, viral titers dropped by 1–2 log at 48–72 h pi. This could be due to (i) cell death (if most cells die, replication stops, and viral titer drops), (ii) virus stability (even though stable environmentally, some viruses may degrade in cell culture medium due to enzymes, temperature, or pH fluctuations that differ from the environmental conditions), and (iii) aggregation or absorption to cell debris or most likely a combination of all.

In NE49CHt-Ep cultures, the magnitude and temporal progression of CPE were comparable among all ReCV isolates, ultimately resulting in complete cell death by 72 hpi (Fig. 6C). Viral titers in NE49CHt-Ep cultures increased progressively over time and reached their peak at 72 hpi, consistent with the replication kinetics observed in enteroid monolayers and LLC-MK2 cells (Fig. 6D). These findings indicate that the conditions in the duodenum-derived cell line are particularly favorable for robust ReCV replication.

### Polarization and barrier function of LLC-MK2 and enteroid-derived cell lines

**Fig 7:**
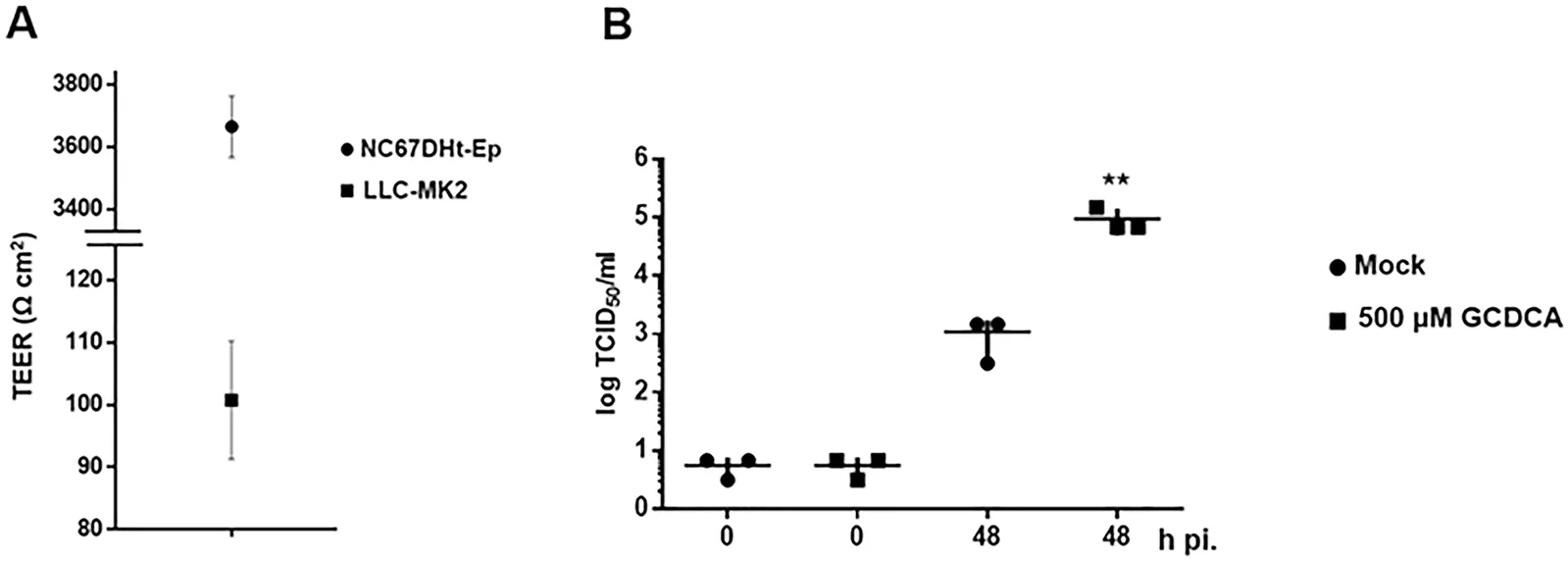
Evaluation of barrier function of enteroid-derived cell lines and the effect of bile on ReCV replication. (**A**) NC67DHt-Ep and LLC-MK2 monolayers were cultured on transwell membranes (0.4 μm; Sarstedt Inc.), and transepithelial electrical resistance (TEER) was measured using an EVOM^2^ epithelial voltohm meter (World Precision Instruments). TEER values for NC67DHt-Ep are shown at 14 days post-seeding, and values for LLC-MK2 monolayers are shown at 6 days post-seeding. (**B**) NC67D enteroid monolayers were infected with ReCV-FT285 at a multiplicity of infection (MOI) of 0.1, with or without GCDCA supplementation. Viral titers were determined by titration in LLC-MK2 cells. Data represent the mean ± SD of three biological replicates. *P* ≤ 0.05; *P* ≤ 0.01; **P ≤ 0.001.

The intestinal epithelium is a highly polarized cellular barrier characterized by extensive tight-junction formation and high barrier integrity. To determine whether the enteroid-derived cell lines recapitulate these features, we measured transepithelial electrical resistance (TEER) in NC67DHt-Ep and NE49CHt-Ep monolayers cultured on Transwell membranes and compared these values with those of LLC-MK2 monolayers (Fig. 7A).

In both intestinal cell lines, TEER stabilized at 12–14 days post-seeding at values exceeding 3,000 Ω·cm², indicating the formation of highly polarized monolayers with exceptionally strong barrier function. These monolayers remained intact for at least 21 days.

In contrast, LLC-MK2 monolayers exhibited substantially lower TEER, never exceeding 120 Ω·cm² under our culture conditions, and began to lose adhesion to the Transwell membrane by 7–8 days post-seeding, indicating limited polarization and barrier-forming phenotype of this cell line.

Collectively, these findings demonstrate that rhesus enteroids and enteroid-derived epithelial cell lines more closely recapitulate the polarized, tight-junctioned architecture and high barrier integrity of the native intestinal epithelium than LLC-MK2 cells. Consequently, they provide a substantially more physiologically relevant model for enteric calicivirus research, enabling studies of viral infection, entry, and intestinal epithelial barrier crossing.

### Effect of bile on ReCV replication

Most HuNoVs require bile for efficient replication in enteroid monolayers. However, our results demonstrate that ReCV can replicate robustly in enteroid monolayers even in the absence of bile. Similarly, GII.4 HuNoVs are capable of replicating without bile, although their replication is significantly enhanced by bile supplementation. To further investigate the impact of bile on ReCV replication, highly differentiated NC67DHt-Ep monolayers were infected with ReCV-FT285 at an MOI of 0.1, either with or without supplementation with 500 μM GCDCA. At 48 h post-infection, cultures supplemented with GCDCA showed a significant increase in ReCV titers compared to untreated controls, indicating that bile enhances ReCV replication under these conditions (Fig. 7A).

## DISCUSSION

HuNoV research has long been hindered by the absence of a robust *in vitro* propagation system, necessitating reliance on cell culture-propagable surrogate caliciviruses. The introduction of the enteroid culture system represented a major breakthrough for HuNoV studies; however, important limitations remain, and surrogate viruses continue to serve as indispensable experimental tools. Among these, ReCVs are biologically the closest to HuNoVs, and their use has substantially advanced calicivirus research, particularly in food and environmental virology. Here, we sought to further enhance the utility of the ReCV surrogate system by developing a physiologically relevant rhesus macaque enteroid–based culture model that closely parallels HuNoV enteroid systems.

We report the establishment of multiple intestinal stem cell-derived enteroid lines from several individual rhesus macaques and distinct regions of the gastrointestinal tract. We demonstrated that these enteroids contained all major intestinal cell types described in other systems, could be readily expanded, cryopreserved in liquid nitrogen, and successfully revived.

One unexpected observation was the notably high expression of sucrase-isomaltase in the NC67C enteroid (Fig. 2B). Sucrase-isomaltase is a mucosal disaccharidase normally restricted to the brush border of enterocytes in the healthy small intestine. While it is expressed in the developing fetal colon, sucrase-isomaltase is generally absent from adult colonocytes. However, ectopic sucrase-isomaltase expressions have been reported in colonic neoplasia, such as adenocarcinomas, as well as in colitis-associated dysplasia (17–19). In this case, the donor animal was an infant euthanized due to chronic colitis, a condition often associated with epithelial remodeling and metaplasia, whereby colonic epithelium adopts features of a small intestinal phenotype. Additionally, stem cells within enteroid cultures may lose their regional identity under suboptimal conditions (e.g., high levels of Wnt or R-spondin), potentially leading to aberrant expression of sucrase-isomaltase.

Infection studies with three biologically distinct ReCV strains in enteroid monolayers revealed several key differences between ReCVs and HuNoVs. Notably, ReCVs exhibited markedly high levels of viral replication, greater viral yields, pronounced cytopathic effects, and the ability to replicate in colon-derived enteroids—features not observed for HuNoVs. Furthermore, while most HuNoV strains (except GII.4) require bile for replication in enteroid monolayers, all three ReCV strains tested in this study replicated efficiently in the absence of bile supplementation. Interestingly, as with GII.4 HuNoVs, bile was not required for ReCV replication but significantly enhanced viral yield (Fig. 7B). This finding further underscores the biological similarities between HuNoVs and ReCVs.

Another notable limitation of the HuNoV enteroid system is the difficulty in achieving serial passaging. Although serial passaging of a few HuNoV strains has recently been reported, it remains technically challenging, strain dependent, and considerably less robust compared to many other viruses (10, 11).

In contrast, in our study, all three ReCV isolates were readily serially passaged in enteroid monolayers for at least six passages without any decline in infectious virus titers or the need to suppress innate immune responses (Fig. 3F). This highlights an important distinction between HuNoVs and ReCVs and suggests differences in their innate immune evasion strategies that warrant further investigation.

In addition to enteroid cultures, we established two stable rhesus macaque enteroid-derived cell lines, one from duodenum and the other from colon.

Both cell lines exhibit epithelial-like morphology that remained stable during serial passaging (Fig. 4A). Epithelial-to-mesenchymal transition (EMT is a key biological process in embryonic development and tumor progression, during which epithelial cells lose polarity and cell-to-cell adhesion while acquiring mesenchymal, fibroblast-like properties such as increased motility. In cell culture systems, signaling molecules such as TGF-β are potent inducers of EMT, driving changes in cell morphology, loss of epithelial markers and junctional proteins (e.g., E-cadherin and EpCAM), and acquisition of mesenchymal markers such as vimentin (20).

Characterization of both cell lines confirmed the absence of EMT and the presence of multiple intestinal epithelial cell types. Specifically, the duodenum-derived line contained absorptive enterocytes and enteroendocrine cells, whereas the colon-derived line comprised goblet cells and enteroendocrine cells (Fig. 4A and 5). Together, these observations suggest that both rhesus intestinal cell lines have a mixed cellular composition. This distinguishes them from many classical models, which typically represent a single lineage or a tumor-derived phenotype.

Both cell lines supported robust ReCV infection, yielding high viral titers accompanied by clear cytopathic effects. Notably, while TV and ReCV-FT285 infection resulted in a rapid, complete destruction of the NC67DHt-Ep cell monolayer, ReCV-FT7 infection did not, despite producing similarly high endpoint titers at 24 hpi (Fig. 6). The basis for this difference remains unclear; however, it might suggest that ReCV-FT7 progeny virions may not rely on cell lysis for release in this cell line. Consistent with this idea, we previously reported similar non-lytic yet productive ReCV-FT7 infection in recombinant CHO cells, with suspected exosome-mediated egress of cloaked virions (21). One key difference among these viruses is their attachment mechanism: TV and ReCV-FT285 utilize type B histo-blood group antigens, whereas ReCV-FT7 attachment is dependent on sialic acid (13). Enrichment and characterization of the surviving cell population may help clarify the underlying mechanism.

We did not attempt to identify infected cell types by co-immunostaining infected enteroids for cell type-specific markers and ReCV structural or non-structural proteins or dsRNA, primarily due to the lack of high-quality ReCV-specific antibodies. Moreover, this approach would likely have limited sensitivity for detecting rare or low-abundance cell populations. However, given the widespread lysis of the enteroid-derived cell lines following infection in most cases, it is reasonable to infer that major epithelial cell types present in these cultures, including absorptive enterocytes, enteroendocrine cells, and goblet cells, are capable of supporting infection. Alternatively, the observed complete cell death may reflect rapid infection of a susceptible cell population, followed by activation of innate immune signaling pathways that induce apoptosis in neighboring, uninfected cell types. To address these limitations, we plan to employ single-cell RNA sequencing to identify target cell type(s) in enteroids and enteroid-derived cell lines in a more comprehensive and unbiased manner.

LLC-MK2 cells do not robustly polarize and exhibit weak barrier function. In contrast, both enteroid-derived cell lines formed highly polarized monolayers with exceptionally strong barrier integrity, closely resembling the polarized, tight-junctioned structure of the healthy intestinal epithelium (Fig. 7A). The architecture, cell types, and molecular pathways present in enteroid-derived monolayers enable more realistic modeling of enteric virus infection, entry, and crossing of the epithelial barrier, which is not possible in LLC-MK2 cells due to their kidney origin and lack of intestinal features.

While the exact cell tropism of HuNoVs is still debated, both differentiated enterocytes and enteroendocrine cells have been implicated as target cells, with enterocytes considered the primary targets and enteroendocrine cells secondary targets (5, 22). However, these observations may be influenced by differences between *in vivo* infection and organoid-based systems, as well as by the relative abundance of these cell types in each context, which can bias interpretations of viral tropism. In addition, B cells have also been proposed as potential targets (23–25).

Given that both natural and experimental human norovirus infections in rhesus macaques have been reported by us and others (26–29), these enteroids and derived cell lines may also have direct utility for HuNoV propagation, which is currently under investigation.

In summary, we report the establishment and characterization of multiple rhesus macaque-derived enteroid lines and two enteroid monolayer-derived cell lines. We demonstrated that these culture systems are physiologically relevant and superior to the current ReCV cell culture (LLC-MK2) and significantly improve the ReCV surrogate system by closely aligning with the HuNoV enteroid model.

## MATERIALS AND METHODS

### Tissue samples

Fresh intestinal tissues were collected from rhesus macaques assigned for euthanasia at the Tulane National Primate Research Center, LA (TNPRC), or at the Southwest National Primate Research Center, TX (SNPRC). Animals included both sexes and ages ranging from 7 months to 18 years old (Table 1). A 2 × 2 cm tissue section was collected by the pathologist, placed in a 50 mL tube filled with ice-cold PBS supplemented with 50 µg/mL gentamicin, and thoroughly washed by shaking the tube several times, discarding the fluid, and replacing the PBS until the PBS remained clear. Washed tissues were transferred to fresh 15-mL tubes filled with growth factor-negative culture medium (CMGF−) supplemented with 10 µM Y-27632 (ROCK inhibitor) and 1× penicillin/streptomycin/amphotericin B. Samples were placed on ice and transported to the laboratory. All animal work has been approved by the corresponding institutions’ IACUC.

### Virus stocks and viral vectors

LLC-MK2 cell monolayers grown in M199 medium supplemented with 10% FBS and penicillin/streptomycin/amphotericin B were infected with 0.1 MOI ReCVs at 90% confluency. Cultures were harvested at 48 h post-infection and freeze-thaw to release cell-associated virus. Cell debris was removed by centrifugation, and supernatant was aliquoted and stored at −80°C. Virus stocks were titrated, and titers were recorded (TCID_50_/mL). Three GI ReCV strains—Tulane virus, ReCV-FT7, and ReCV-FT285—representing distinct genogroups, serotypes, and HBGA binding types were used in this study (11).

Lentivirus vector expressing hTERT (Applied Biological Materials, Richmond, BC, Canada) was used for primary cell immortalization.

All work with non-human primate samples and lentivirus vectors has been performed under BSL-2 conditions according to IBC-approved protocols.

### Antibodies

Mouse anti-EpCAM (sc-66020), mouse anti-sucrase isomaltase (sc-393470), mouse anti-vimentin (sc-6260), mouse anti-CAR (sc-70493), mouse anti-E-cadherin (sc-71008), mouse anti-lysozyme C (sc-518012), mouse anti-Muc2 (sc-515032), mouse anti-chromogranin A (sc393941), mouse anti-villin (sc-58897), mouse anti-LGR5 (sc-517661), and mouse anti-GAPDH (sc-47724) monoclonal antibodies (IgG) were purchased from Santa Cruz Biotechnology. In addition, a second mouse anti-chromogranin A monoclonal antibody (MA5-13096, Invitrogen) was obtained.

Anti-A (ABO1) and anti-B (ABO2) mouse monoclonal antibodies (IgM) were purchased from Diagast.

Alexa Fluor 568 goat anti-mouse IgG and IgM Cross-Adsorbed secondary antibodies and horseradish peroxidase (HRP)-conjugated secondary antibodies were purchased from Thermo Fisher Scientific.

### Growth factor-negative culture medium

CMGF− consisted of advanced DMEM/F12 supplemented with 10% FBS, GlutaMAX, HEPES, and penicillin/streptomycin/amphotericin B.

### Growth factor-positive culture medium

Growth factor-positive culture medium (CMGF+) was prepared by mixing equal volumes of CMGF– and L-WRN conditioned medium and supplemented with 1× B27, 1× N2, 10 mM nicotinamide, 1 mM N-acetylcysteine, 50 ng/mL human EGF, 10 nM human [Leu¹⁵]-gastrin I, 500 nM A83-01 (TGFβ pathway inhibitor), 10 µM SB202190 (MAPK inhibitor), 10 µM Y-27632 (ROCK inhibitor).

### Differentiation medium

Differentiation medium consisted of CMGF– medium supplemented with 10% CMGF+ medium.

### L-WRN conditioned medium

The L-WRN conditioned medium, which contains Wnt3a, R-spondin, and Noggin, was prepared in-house. L-WRN cells (ATCC, CRL-3276) were cultured in CMGF– medium, and the culture medium was collected every 2 days for the first 4 days, then daily from day 5 to 7. Cell debris was removed by centrifugation, the medium was filtered (0.2 µm), and stored in aliquots at −80°C.

### Crypt isolation

Pre-cleaned, longitudinally cut intestinal segments were placed into sterile Petri dishes and overlaid with 3–5 mL of chilled rinse buffer (5.6 mM Na_2_HPO_4_, 8.0 mM KH_2_PO_4_, 96.2 mM NaCl, 1.6 mM KCl, 43.4 mM sucrose, 54.9 mM D-sorbitol, pH 7.0). Using sterile scissors and forceps, the tissue was minced into small fragments and transferred into a 15 mL conical tube containing 5 mL of rinse buffer.

The tubes were sealed and vigorously shaken by hand for 20 s. Tissue fragments were allowed to settle, and the supernatant was carefully discarded. This wash was repeated five times, transferring the tissue into a fresh 15 mL tube after every second wash.

Following the final wash, the tissue was resuspended in 2 mL of rinse buffer supplemented with 20 mM EDTA (chelating buffer) and transferred to a well of a six-well tissue culture plate. The plate was placed on an orbital shaker at 250 rpm at 4°C. Tissue samples were monitored every 10 min under a light microscope for the release of intestinal crypts.

Once a significant number of free-floating crypts were observed, the tissue and rinse buffer were transferred to a 15 mL tube containing 5 mL of rinse buffer supplemented with 20% FBS. The mixture was pipetted up and down 10–15 times using a transfer pipette. Tubes were then placed on ice to allow tissue fragments to settle.

The supernatant, containing released crypts, was transferred to a new tube and centrifuged at 150 × *g* for 5 min at 4°C. The resulting crypt pellet was resuspended in 2 mL of CMGF– medium containing 10 μM Y-27632. A 10 μL aliquot was removed to count crypts under a microscope.

Crypts were then evenly distributed into two Eppendorf tubes (1 mL each) and pelleted at 100 × *g* for 5 min at 4°C. Based on the crypt count, Matrigel was added to achieve a final concentration of 100–150 crypts per 20 μL of Matrigel. The crypt-Matrigel suspension was gently mixed using a P200 pipette tip, and 20 μL of the mixture was plated per dome into the wells of a 12-well tissue culture plate (3–4 domes per well).

Plates were incubated upside-down at 37°C in a 5% CO_2_ incubator for 10 min to allow Matrigel polymerization. Afterward, each well was overlaid with 1 mL of pre-warmed CMGF+ medium. Cultures were observed daily under a light microscope, and medium was refreshed every 2–3 days. Enteroid development was monitored, and cultures were passaged every 6–9 days.

### Enteroid passage

Culture medium was removed from wells containing Matrigel domes with mature enteroids. Wells were rinsed with 1 mL of ice-cold DPBS and overlaid with 0.5 mL of ice-cold cell recovery solution (Corning, Cat. #354253). Plates were wrapped in aluminum foil and incubated at 4°C for 30–45 min. Domes were dislodged using a P1000 pipette with a filter tip and gently pipetted up and down until most of the Matrigel had dissolved. The contents of each well were transferred to sterile microcentrifuge tubes (up to two wells per tube) and kept on ice for 10 min. If residual Matrigel remained in the wells, an additional 0.5 mL of cell recovery solution was used to rinse the wells, and the wash was added to the corresponding tubes.

Enteroids were collected by centrifugation (500 × *g*, 4°C, 5 min) and resuspended in 1 mL of CMGF− medium supplemented with 10 µM ROCK inhibitor. Mechanical dissociation was performed using a 1 mL syringe fitted with a 25G × 5/8″ needle by aspirating and dispensing the suspension 2–3 times per tube. Enteroids were collected again by centrifugation, and the supernatant was carefully removed. Pellets were resuspended in Matrigel at a volume of 1.5–2 × (the number of original domes per tube × 20 µL) and plated as 20 µL domes on pre-warmed 12-well plates (3–4 domes per well). Plates were incubated upside-down at 37°C in a 5% CO_2_ incubator for 10 min to allow Matrigel polymerization, then overlaid with 1 mL of pre-warmed CMGF+ medium.

Culture medium was refreshed every 2–3 days, and enteroids were passaged every 6–9 days.

### Enteroid cryopreservation

Enteroids were harvested and released from Matrigel as described above, then resuspended in 1 mL of Recovery cell culture freezing medium (Gibco) per enteroid preparation (derived from 2 to 3 original domes). The suspension was transferred to cryovials and placed in a controlled-rate freezing container at –80°C overnight. The following day, the cryovials were transferred to liquid nitrogen for long-term storage.

### Enteroid monolayer preparation

Enteroids were recovered from Matrigel as described above, rinsed with DPBS, and pooled from approximately 10–15 domes. The collected enteroids were resuspended in 0.5 mL of 0.05% trypsin/0.5 mM EDTA solution and transferred to a 12-well tissue culture plate. Samples were incubated at 37°C in a 5% CO_2_ incubator for 5 min. Trypsin activity was then neutralized by adding 1 mL of CMGF– medium supplemented with 10 μM Y-27632 (ROCK inhibitor).

Trypsinized enteroids were gently dissociated by passing the suspension three times through a 1 mL syringe fitted with a 25 G × 5/8″ needle. The resulting single-cell suspension was examined under a light microscope to assess dissociation efficiency, transferred to microcentrifuge tubes, and centrifuged to collect cells. The cell pellet was resuspended in CMGF+ medium. If large cell clumps were still visible, the suspension was allowed to settle by gravity for 5–10 min, and the upper phase containing dissociated cells was collected for counting. Larger aggregates were either re-trypsinized or re-plated in Matrigel for further culture.

Cells were seeded at a density of at least 1 × 10^5^ cells per well in collagen-coated 96-well plates. Cultures were maintained in CMGF+ medium for 2 days, after which the medium was replaced with differentiation medium (DM). Cells were cultured in DM for an additional 4–6 days, with medium changes every 2 days, prior to use in infection assays.

### Immortalization of rhesus macaque enteroid monolayers

Enteroid monolayer cultures were prepared by seeding single-cell suspensions derived from enteroids onto collagen-coated 96- or 48-well tissue culture plates. On day 2, the CMGF+ medium was replaced with DM. Monolayers were cultured until reaching approximately 50%–60% confluency and transduced with a lentiviral vector carrying hTERT at an MOI of 5, following the manufacturer’s transduction protocol using polybrene. Medium was refreshed every 3 days, and monolayers were passaged every 9–10 days using TrypLE Express reagent (Thermo Fisher Scientific), with surviving cells re-seeded onto collagen-coated plates. Passaging continued until surviving cells could be expanded to a six-well plate and further to T25 flasks. At that stage, the majority of cells were used to establish cryopreserved backup stocks. Mock transduced cultures served as controls and were processed in parallel.

### Virus titration

LLC-MK2 cells (1 × 10⁴ cells per well) were seeded in 96-well plates and inoculated with 10-fold serial dilutions of the virus samples (100 μL per well; three wells per dilution). Plates were incubated for 5 days, after which cells were fixed and stained with crystal violet. Viral titers (TCID_50_) were determined using the Reed and Muench method.

### Immunofluorescence staining

Cell monolayers were grown directly on chamber slides. Enteroids grown in Matrigel domes were recovered when morphology indicated differentiation (day 6–7) using cell recovery solution, washed with ice-cold PBS, and plated onto Matrigel-coated chamber slides in CMGF+. Chamber slides were incubated at 37°C for 4 h to allow the enteroids to settle onto the Matrigel layer. The medium was then removed by gentle aspiration, and the slides were rinsed with PBS, fixed with 4% paraformaldehyde, and permeabilized with 0.1% Triton X-100 for 10–15 min. Slides were then blocked with PBS containing 5% goat serum for 2 h at room temperature. Primary antibodies diluted in PBS—5% goat serum—0.1% Tween 20, added to wells of chamber slides, and slides were incubated overnight at 4°C. The following day, slides were washed three times with PBS—0.1% Tween 20 (PBS-T, pH 7.4) at room temperature. Secondary antibodies, diluted in PBS—5% goat serum—0.1% Tween 20 were applied, and slides were incubated for 1.5 h at room temperature in the dark. After three washes, slides were mounted with ProLong Diamond antifade mountant containing DAPI (ThermoFisher Scientific) and imaged using an EVOS FLoid cell imaging station (Life Technologies).

### Western blot immunostaining

Enteroids grown in Matrigel domes were recovered from four domes on day 6 using cell recovery solution and washed with ice-cold PBS. Samples were then lysed in 0.3 mL of Pierce RIPA buffer (Thermo Fisher Scientific) supplemented with Halt protease inhibitor cocktail (Thermo Fisher Scientific). Lysates were incubated on ice for 30 min with vortexing every 5 min. Insoluble material was removed by centrifugation at 12,000 rpm for 15 min at 4°C. The resulting supernatants were aliquoted and stored at −80°C until further use. Protein concentrations were determined using the Bradford protein assay (Bio-Rad).

For analysis, samples (~20 µg total protein) were mixed with an equal volume of 2× Laemmli sample buffer (Bio-Rad), boiled for 5 min, separated on 4%–20% Mini-PROTEAN TGX precast gels (Bio-Rad), and transferred to polyvinylidene difluoride membranes (Bio-Rad). Membranes were blocked with 5% nonfat dry milk in PBS-T for 3 h at room temperature, rinsed with PBS-T, overlayed with primary antibodies (0.25 µg/mL) diluted in blocking buffer, and incubated overnight at 4°C on a rotary shaker. After three washes with PBS-T, membranes were incubated with HRP-conjugated secondary antibodies for 3 h at room temperature, washed three times, and developed using SuperSignal West Femto Maximum Sensitivity substrate (Thermo Fisher Scientific). Signals were visualized using a ChemiDoc MP Imaging System (Bio-Rad). In some cases, membranes were stripped using Restore PLUS Western Blot Stripping Buffer (Thermo Fisher Scientific) and re-probed.

### Confocal microscopy

Confocal images were acquired using an Olympus FV4000 laser-scanning confocal microscope equipped with a 60× oil-immersion objective. Maximum-intensity projections of *z*-stack images were generated. Representative fluorescent image stacks were also reconstructed in three dimensions for 3D visualization.

### Statistical analysis

Experiments were performed in at least three independent replicates. Differences between virus titers were evaluated for statistical significance by an unpaired *t*-test using the GraphPad software (Dotmatics). A *P*-value ≤ 0.05 was considered statistically significant.

## Data Availability

The data that support the findings of this study are available upon reasonable request to the corresponding author.
